# Temozolomide and Pituitary Tumors: Current Understanding, Unresolved Issues, and Future Directions

**DOI:** 10.3389/fendo.2018.00318

**Published:** 2018-06-15

**Authors:** Luis V. Syro, Fabio Rotondo, Mauricio Camargo, Leon D. Ortiz, Carlos A. Serna, Kalman Kovacs

**Affiliations:** ^1^Department of Neurosurgery, Hospital Pablo Tobon Uribe and Clinica Medellin, Medellin, Colombia; ^2^Department of Laboratory Medicine, Division of Pathology, St. Michael’s Hospital, University of Toronto, Toronto, ON, Canada; ^3^Genetics, Regeneration and Cancer Laboratory, Universidad de Antioquia, Medellin, Colombia; ^4^Division of Neuro-oncology, Instituto de Cancerología, Clinica Las Americas, Pharmacogenomics, Universidad CES, Medellin, Colombia; ^5^Laboratorio de Patologia y Citologia Rodrigo Restrepo, Department of Pathology, Clinica Las Américas, Universidad CES, Medellin, Colombia

**Keywords:** alkylating agents, chemotherapy, DNA repair, neoplasms, neuroendocrine tumors, O(6)-Methylguanine-DNA Methyltransferase, pituitary, temozolomide

## Abstract

Temozolomide, an alkylating agent, initially used in the treatment of gliomas was expanded to include pituitary tumors in 2006. After 12 years of use, temozolomide has shown a notable advancement in pituitary tumor treatment with a remarkable improvement rate in the 5-year overall survival and 5-year progression-free survival in both aggressive pituitary adenomas and pituitary carcinomas. In this paper, we review the mechanism of action of temozolomide as alkylating agent, its interaction with deoxyribonucleic acid repair systems, therapeutic effects in pituitary tumors, unresolved issues, and future directions relating to new possibilities of targeted therapy.

## Introduction

Since its development, temozolomide (TMZ), a monofunctional alkylating agent, has shown remarkable efficacy in the treatment of a variety of solid tumors and it has become an essential component of adjuvant therapy for the most frequent adult brain tumor type, glioblastoma multiforme (GBM) (1, 2). Its unique chemical structure and pharmacokinetic properties confer distinctive advantages over other alkylating agents. In 2006, TMZ began to be used for the treatment of aggressive pituitary adenomas and pituitary carcinomas (3–5). In this paper, we review the mechanism of action of TMZ, the deoxyribonucleic acid (DNA) repair systems triggered after its administration, the current understanding of TMZ activity, and the unresolved issues of its clinical use in pituitary tumors.

## Alkylating Agents

During World War I, alkylating agents were used as chemical weapons. Sulfur mustard, commonly known as mustard gas, when released into the air was a threat to skin and mucous membranes. Its vesicant effect caused large blisters on exposed skin, eye irritation, upper and lower airway inflammatory reactions, bone marrow aplasia, and pancytopenia (6). Even the slightly less toxic nitrogen mustards also exhibited cytotoxic effects causing bone marrow suppression after exposure and subsequent absorption into the body. In the 1940s, given their ability to produce significant tumor regression, alkylating agents became the earliest types of drugs used to treat cancer. The first clinical use as a chemotherapeutic agent was performed in 1942 but it was not reported for several years (7).

Alkylating agents are ubiquitous, highly reactive compounds that transfer alkyl radicals into a broad range of biologically active nucleophilic molecules (8). They are electrophilic compounds (behave as electron acceptors) that react with nucleophilic moieties (behave as electron donors) of DNA or proteins. This results in the covalent transfer of an alkyl group (from the alkylating agent) to the DNA or the protein (9). The alkyl substituents are acyclic, saturated, hydrocarbon chains that have lost one hydrogen. The main cellular target of alkylation is DNA, and cytotoxicity is due to the alkylation of its bases which impairs its function as a template during replication and transcription (9). Alkylating agents are a diverse group of chemical substances. Due to their unique properties, they can be genotoxic, but may be used in the treatment of tumors as well (8).

Alkylating agents can be mono- or bifunctional. Monofunctional agents have a single reactive group that interacts with a single center in DNA, whereas bifunctional agents have two groups which can react with two different sites in DNA (10, 11). Consequently, one monofunctional alkylating molecule produces a covalent adduct on one base only but with bifunctional alkylating agents, alkylation can occur on two bases within the same DNA strand (intrastrand crosslinking), or from opposite strands (interstrand crosslinking) (10, 12, 13).

**Figure 1:**
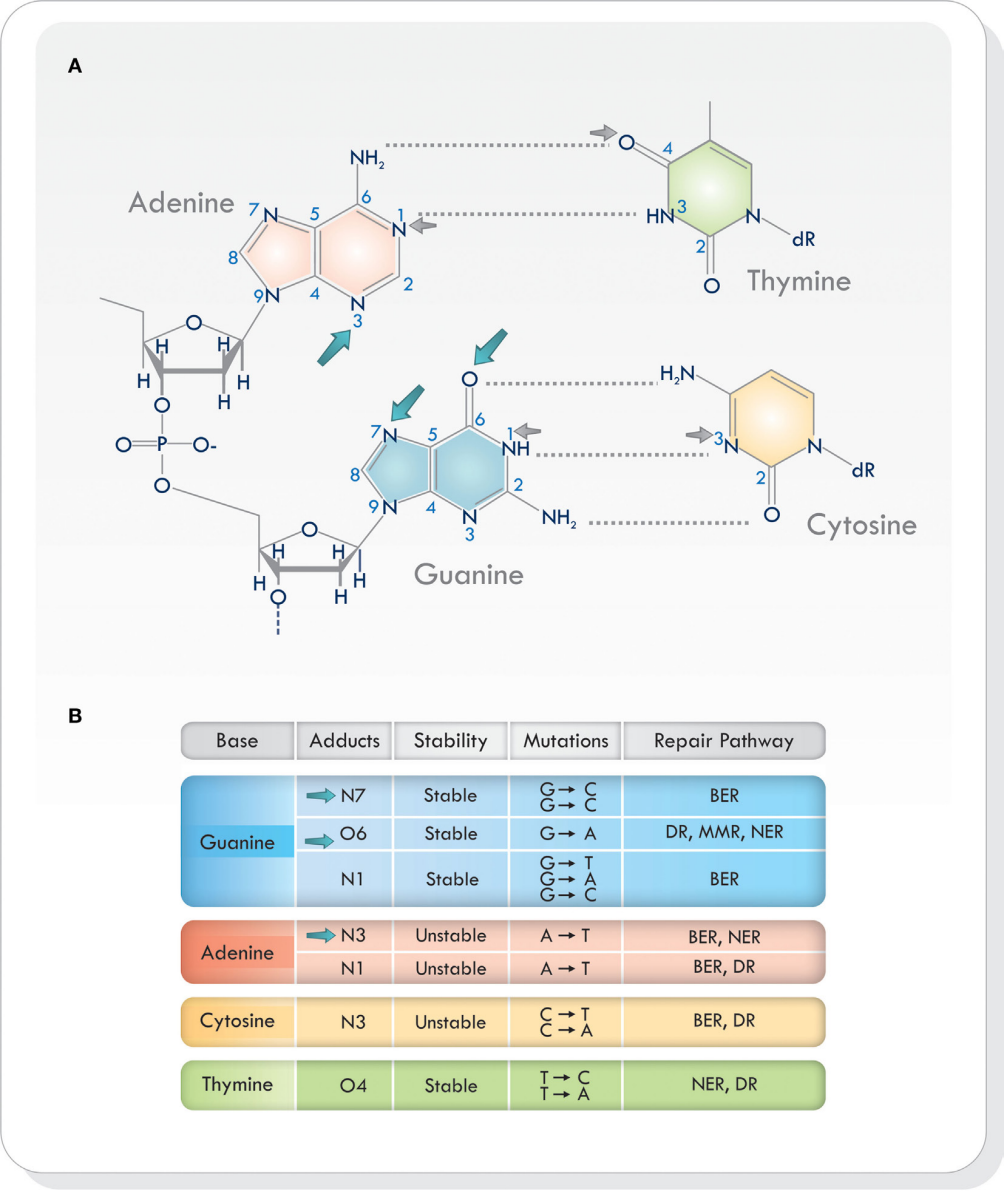
**(A)** Sites of alkylation on the various bases in the DNA. Temozolomide produces alkylation on position N7 and O6 in guanine and N3 in adenine (blue arrows). Other alkylating agents act on different sites of alkylation (small gray arrows). **(B)** Chemical stability, mutagenesis, cytotoxicity, and repair mechanisms in different sites of alkylation. Based on Ref. (8, 9, 16, 19). Abbreviations: BER, base excision repair; DR, direct repair; MMR, mismatch repair; NER, nucleotide excision repair; DNA, deoxyribonucleic acid.

Virtually all heteroatoms (non-carbon or hydrogen atoms) in DNA can be alkylated and, depending on the nucleophile and the alkylating agent, there are different sites of alkylation in double-stranded DNA (9, 12, 14–16). Base alkylation occurs mainly on position N7 and O6 in guanine (N7-MeG and O6-MeG), N1 and N3 in adenine (N1-MeA and N3-MeA), and N3 in cytosine (N3-MeC) (9) (Figure 1A). Additional alkylation sites do arise but are less frequent. Every site of alkylation has different consequences in chemical stability, mutagenesis, and cytotoxicity (8, 9). DNA adducts O6-MeG, N1 in guanine (N1-MeG), N7-MeG and O4 in thymine (O4-MeT) are both stable and mutagenic, whereas alkylation on other sites (i.e., at certain endocyclic nitrogen) yield chemically unstable adducts, making the purine/pyrimidine base-rings in the DNA susceptible to hydrolytic attack and base-ring opening (9, 15) (Figure 1B). Because alkylating agents produce more than one type of adduct, the exact consequence of base alkylation on the function of the cell is difficult to evaluate (9, 15). From the various types of DNA adducts produced, O6-MeG, which is the least frequent (5–10%), is the most cytotoxic lesion (17, 18).

Alkylating agents were the first chemotherapeutic anti-cancer agents developed and are the largest group of drugs among cytotoxic chemotherapeutics (10). They consist of three different groups: classical, non-classical, and alkylating-like agents. Many alkylating agents are categorized as “classical” alkylating agents. They include true alkyl groups. Alkylating-like agents are platinum-based analogs and do not have an alkyl group. They can also interfere with DNA repair and can permanently damage it; hence, they are referred to as “alkylating-like.” The third group is known as “non-classical.” To date, there is no consensus on which agents belong to this category. Most classical alkylating agents and alkylating-like agents cause cytotoxicity by inducing DNA crosslinking while non-classical alkylating agents such as dacarbazine, procarbazine, and TMZ, induce O6-MeG, N7-MeG, N3-MeA, without triggering DNA crosslinking because they are monofunctional (10).

## Temozolomide

**Figure 2:**
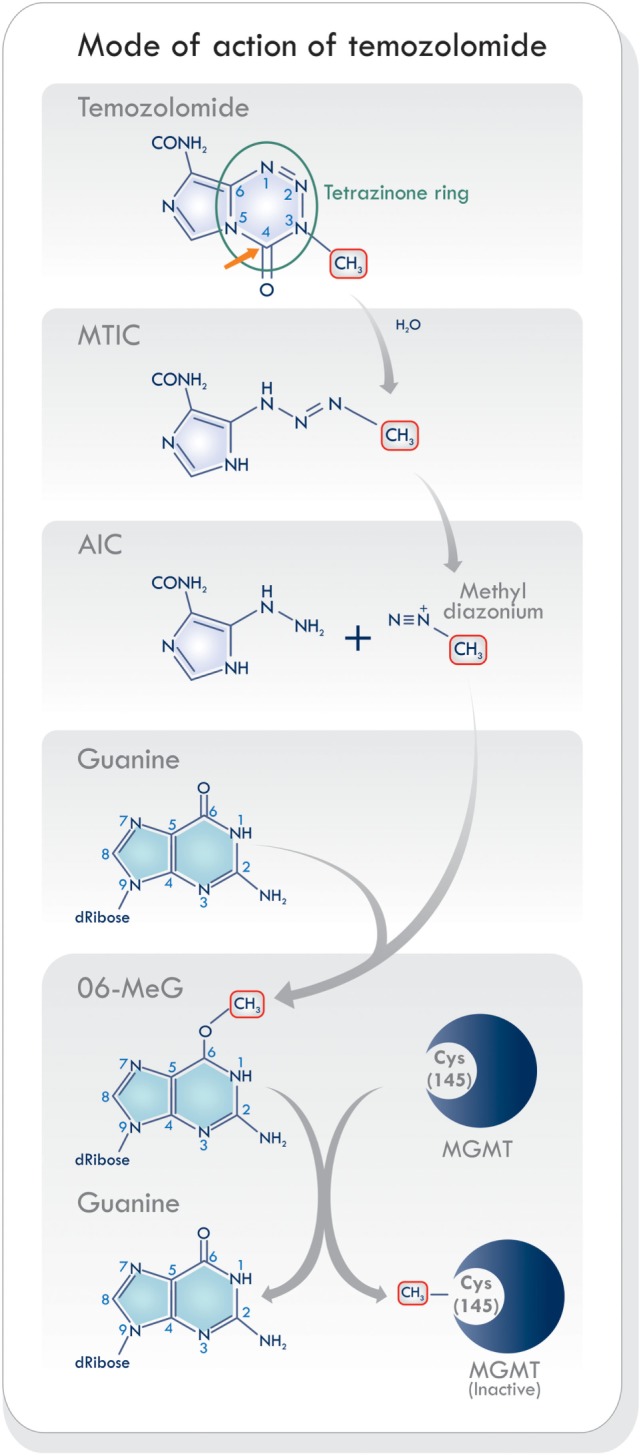
Schematic of mode of action of temozolomide (TMZ). The activation of TMZ in physiologic pH levels within the blood stream into its reactive state that is responsible for methylating DNA. Based on Ref. (8, 19, 23). Abbreviations: MTIC, 5-(3-monomethyl-1-triazeno) imidazole-4-carboxamide; AIC, 5-aminoimidazole-4-carboxamide; MGMT, O6-methylguanine-DNA methyltransferase.

Temozolomide is a monofunctional alkylating agent belonging to the triazene group of non-classical alkylating agents, characterized by the presence of three adjacent nitrogen atoms (20) (Figure 2). It is related to a series of imidazotetrazines developed by Stevens and his associates in Birmingham, United Kingdom (21). Temozolomide is a lipophilic, low molecular weight (194 Da) prodrug with no pharmacological activity until it is hydrolyzed. It is orally administered, stable at acidic stomach pH levels, and reactive at pH levels above 7.0 (22). Its activation starts with the hydrolytic cleavage of the tetrazinone ring (Figure 2, green circle). The effect of water at the C4 position of TMZ opens the ring (Figure 2, orange arrow), releases carbon dioxide, and produces 5-(3-monomethyl-1-triazeno) imidazole-4-carboxamide (MTIC) which is an unstable and short-lived active compound. It undergoes further cleavage, to generate 5-aminoimidazole-4-carboxamide (AIC), and the highly reactive methyl diazonium methylating species (2, 23) (Figure 2). This cation methylates DNA producing the adducts N7-MeG (60–80%), N3-MeA (10–20%), and O6-MeG (5–10%). During the next cycle of DNA replication, the sequence of mismatch repair events results in cell cycle arrest which leads to cell death, as will be discussed below. Temozolomide activity increases as its accumulation in tumors rises. It has been shown that brain tumors have a more alkaline pH than healthy tissues, which may favor the activation of TMZ (19, 24). Hence, the pH levels in the microenvironment may be an essential factor modulating its cytotoxic effects and resistance.

## DNA Damage and Repair Mechanisms of Alkylating Agents

Deoxyribonucleic acid can be damaged by external agents or by unwanted products arising from the cell chemistry (14, 15, 25–29). Internal chemical events that threaten DNA stability are depurination, deamination, reactive oxygen species (ROS), and non-enzymatic methylation. In addition, DNA replication has a spontaneous and inevitable error rate that may persist despite proofreading/DNA editing mechanisms (30). External agents that likely cause DNA damage are ionizing and ultraviolet radiation, environmental chemicals, and chemotherapeutic agents such as TMZ.

During DNA replication, various types of damage can cause the replication fork to stall. These events may be potentially lethal to the cell, but complex and specialized multi-protein mechanisms continuously monitor the DNA to detect and repair any damage. If the cell is not able to restore DNA integrity, it may sustain cytotoxic or mutagenic perturbations. *Cytotoxicity* usually arises when progression through the cell cycle is delayed at repair checkpoints; if the damage inflicted to the genome overwhelms the repair capacity of the cell, apoptosis is triggered. By contrast, *mutagenesis* can occur if the cell sustains minor damages in the DNA yet it survives, and after cell division, these unrepaired damages may cause mispairing of bases that result in base-pair substitution which become a consolidated mutational change only after successive DNA replication cycles (8).

There are several mechanisms to correct or prevent alkylation-induced DNA damage. One of them occurs during DNA replication, and relies on the catalytic feature of the DNA polymerases delta and epsilon (Pol δ, Pol ε), which have a 3′ → 5′ exonuclease activity (known as proofreading) (30, 31). When DNA has an alkylated base, a mispairing is generated; subsequently, during DNA synthesis the incorrect base pair is recognized, the direction of DNA polymerase is reversed by one base pair, the mismatched base is excised, and the correct one is inserted. Then, DNA replication can continue forward. Proofreading is only one of several systems to prevent mutations or to repair damaged DNA (15, 25–28, 32, 33).

*Direct repair* (DR) of DNA can be achieved by removing only the abnormal alkyl group, without removing the base or nucleotide. This typically occurs in the G1 phase of the cell cycle. The cell uses this mechanism in the repair of O6-MeG by O6-methylguanine-DNA methyltransferase (MGMT), which removes the methyl group leaving the base intact (9, 15, 32) (Figure 2).

Another group of mechanisms involves excision repair, where a single base, nucleotide, or a segment of the damaged DNA strand is excised, and the gap is filled by a combination of DNA polymerase and ligase. Three well-known excision repair systems used to detect and correct cellular DNA damage include: base excision repair (BER), nucleotide excision repair (NER), and mismatch repair (MMR).

*Base excision repair* includes DNA glycosylases recognition of small distortions in DNA caused by damage to a single base or uracil misincorporation (19). Lesion-specific glycosylases recognize the damaged base, and the *N*-glycosidic bond is hydrolytically cleaved. This generates an internal abasic site with a remaining external phosphodiester backbone which is then cut by a specific endonuclease (AP-endonuclease). The gap is finally repaired by polymerase beta and ligase-III. *Nucleotide excision repair* involves the initial enzymatic recognition of local distortions of the DNA helix caused by mismatched bases or bulky adducts, followed by the removal of a short single-stranded DNA segment containing the lesion. Afterward, DNA polymerase fills the gap by synthesizing the short complementary sequence, completing the repair (9). *Mismatch repair* is a complex system for recognizing and repairing erroneous insertion, deletion, and misincorporation of bases. It involves removal of tens or hundreds of bases at both sides of the damaged base, or of the bases that do not form Watson–Crick base pairing. It is a highly conserved repair pathway in both eukaryotes and prokaryotes (34). Hence, human MMR proteins are homologs to the *E. coli* MMR proteins: MutL homologs 1 and 3 (MLH1 and MLH3), MutS homologs 2, 3, and 6 (MSH2, MSH3, and MSH6), and postmeiotic segregation increased proteins (PMS1 and PMS2). Therefore, the MMR is strand-specific (35). Loss of MMR function results in Lynch Syndrome (OMIM #120435) caused by heterozygous mutations in MMR genes. Although the DR mechanism is mainly involved during the repair of TMZ induced damages, all systems mentioned above do participate.

## DNA Repair and Temozolomide Action

### Direct Repair

Direct repair of the effects of TMZ is carried out by MGMT, a small enzymatic protein (22 kDa) that removes the methyl group from the O6-MeG adduct (19). It is a stable protein with a half-life of more than 24 h. The O6-methyl group is transferred from the guanine to an internal cysteine residue (Cys 145) on the MGMT, in a one-step reaction without relying on cofactors or enzymes (36) (Figure 2). This process removes methyl/alkyl molecules at a 1:1 ratio and restores guanine to its normal structure, thereby eliminating any further DNA strand breaks. In this manner, MGMT acts as an acceptor molecule by removing (sequestering/accepting) the methyl group from the O6-MeG. It then becomes inactivated and degraded through ubiquitination (19). For this reason, it has been called a suicide enzyme (19, 37). Although MGMT protects healthy cells from alkylating carcinogens, it also protects tumoral cells from the same kind of chemical genotoxicity. The non-discriminating effect of MGMT on both healthy and tumoral cells is of concern since it can counteract the effects of TMZ treatment. In some tumors, low-level expression of MGMT may result from epigenetic silencing of its gene. On the contrary, some tumors may display increased MGMT activity when compared to their corresponding healthy tissue (38) thereby presenting an increase in tumor resistance to TMZ treatment. From this perspective, low-level MGMT expression can be considered a favorable predictive marker in TMZ-treated patients (39).

The MGMT-mediated repair system is unique in many aspects, consequently making it a difficult task to target MGMT within tumor cells. The capacity to repair of MGMT depends on the number of active MGMT molecules. If the number of adducts exceeds the preexisting levels of MGMT molecules, the rate of repair will depend on *de novo* synthesis. This process is important since the effect of TMZ depends both on the level of MGMT present within the tumor cells, as well as the number of DNA adducts (2) and so, an excess amount of DNA adducts can completely exhaust the MGMT catalytic levels.

Recently it has been reported that the DNA oxidative demethylase ALKBH2, capable of directly reversing N1-MeA and N3-MeC in DNA, was abundantly expressed in established GBM cell lines and human GBM; and that TMZ exposure increased cellular ALKBH2 expression levels (40). Therefore, these authors propose that ALKBH2 as a novel mediator of TMZ resistance in human GBM.

### Base Excision Repair

**Figure 3:**
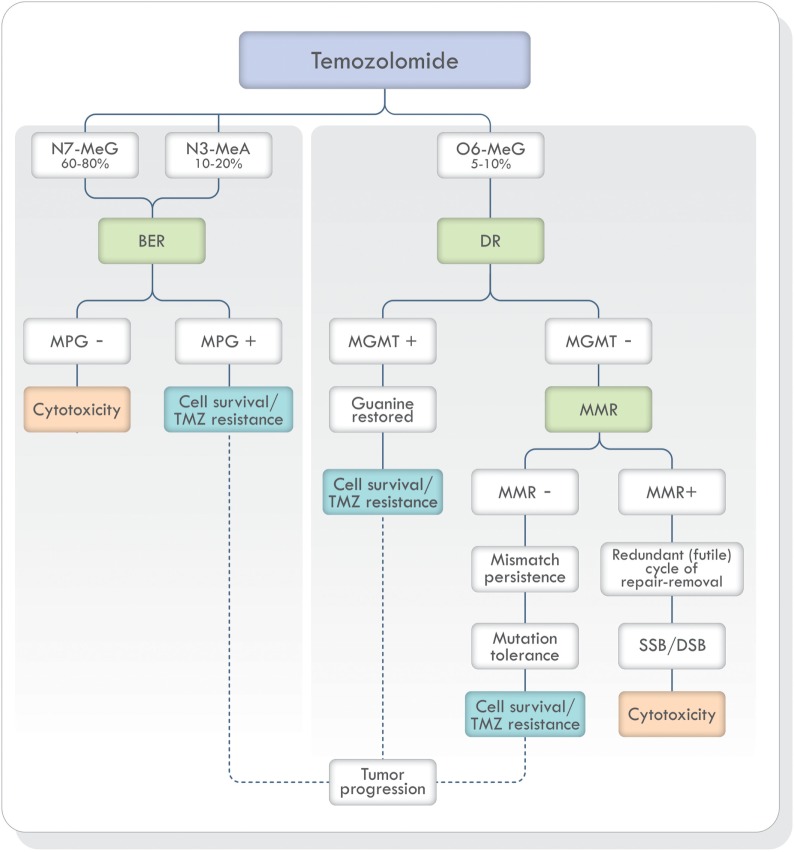
TMZ action and DNA repair mechanisms involved in the prevention and correction of alkylation-induced DNA damage. Based on Ref. (19, 42, 47, 48). Abbreviations: BER, base excision repair; DR, direct repair; MMR, mismatch repair; NER, nucleotide excision repair; MPG, DNA methylpurine*-N*-glycosylase; MGMT, O6-methylguanine-DNA methyltransferase; TMZ, temozolomide; SSB, single-strand breaks; DSB, double-strand breaks; DNA, deoxyribonucleic acid.

Non-bulky modified or damaged bases are removed and repaired by BER (19, 33, 41). As stated earlier, TMZ generates mostly N7-MeG and N3-MeA adducts (90–94%) (Figure 3). These highly toxic adducts are initially removed by DNA methylpurine-*N*-glycosylase (MPG), also known as alkylpurine-DNA–*N*-glycosylase (APNG) (42–44). MPG belongs to one of the four glycosylase families involved in BER (44). Agnihotri et al. (42) showed that APNG, together with MGMT, may provide TMZ resistance in an additive manner since, in TMZ resistant GBM cell lines, they are both expressed. In addition, APNG may be epigenetically regulated due to methylation of the *APNG* gene promoter which attenuates its expression. They also found that GMB patients with nuclear APNG immunostaining in their biopsies had significantly worse overall survival (OS) (42). On the contrary, GBM patients with a methylated *MGMT* promoter and good survival, also had a greater number of APNG-negative tumors, suggesting that low MGMT and low APNG immunoexpression lead to better TMZ response. Based on these findings, they proposed that in GBM patients with methylated *MGMT* promoter, evaluation of APNG immunoexpression would be beneficial (42, 45, 46).

In addition, poly(ADP-ribose) polymerase-1 (PARP-1) is an essential protein for the adequate function of BER. Therefore, if PARP-1 function is disrupted, BER will be inhibited in which case the cytotoxic effects caused by TMZ will be increased (19, 45, 48).

### Mismatch Repair

The adduct O6-MeG produced by TMZ, if not repaired by MGMT, creates a structural distortion of DNA recognized by MMR (Figure 3). Mismatch repair can correct the error by replacing O6-MeG with guanine or leave single-strand breaks which in turn can generate potentially lethal double-strand breaks (DSB) during DNA replication. Depending on the type of damage, stalling at the replication forks during S phase, cell cycle arrest, cytotoxicity, and cell death can occur (49). If, on the other hand, MMR does not recognize the O6-MeG-thymine mispairs, O6-MeG lesions will be tolerated, and the cells will survive (50). Mutagenesis of the O6-MeG adduct can also arise if the original [06-MeG]::[C] mispair remains unrepaired until the next cell cycle and then, pairing with thymine [06-MeG]::[T] instead of cytosine. Subsequent replication cycles, will create a true stable mutation due to the formation of a [A]::[T] pair. In short, the mutagenesis sequence would be [G]::[C], [06-MeG]::[C], [06-MeG]::[T], [A]::[T].

A successful MMR system is therefore required for TMZ cytotoxicity (19, 47, 49) and MMR deficient cells are resistant to TMZ treatment (Figure 3) (19, 51–53). Publications focusing on the mechanisms of acquiring TMZ resistance in pituitary tumors have shown that one patient presented progression and resistance to TMZ with loss of MSH6 protein immunoexpression during treatment (54) and in another publication, a patient with germline *MSH2* mutation was unresponsive to TMZ therapy (55). Therefore, MSH6 and MGMT immunoexpression analysis has been proposed in the morphologic study of pituitary tumors (56–60).

## Aggressive Pituitary Adenomas and Pituitary Carcinomas

Pituitary adenomas are a heterogeneous group of lesions with different clinical behaviors. Although current treatment protocols have been able to control the majority of pituitary adenomas, some of them may recur despite repeated surgeries, radiotherapy, and pharmacologic treatments. These “difficult to treat” tumors have been called *aggressive pituitary adenomas* (61, 62) and their prevalence remains uncertain. According to the current (2017) World Health Organization (WHO) classification of tumors of endocrine organs (58, 59), pituitary adenomas can only be characterized as *pituitary carcinomas* when craniospinal or systemic metastases are found. Pituitary carcinomas are rare and account for approximately 0.12% of pituitary adenomas in the German Pituitary Tumor Registry (63).

The previous (2014) WHO classification characterized a subtype of adenomas as “atypical” if they presented an elevated mitotic index, a Ki-67 labeling index greater than 3%, and extensive nuclear staining for p53 immunoreactivity (64). It was assumed that these atypical adenomas might have an uncertain clinical and biological behavior. This assumption has not been proven and, to date, it also has not been able to accurately predict tumor recurrence or resistance to therapy (65, 66). Thus, in the recent 2017 WHO classification of pituitary adenomas, the term “atypical adenomas” is no longer recommended (59, 67, 68).

In the past, the terms atypical, invasive, and aggressive have been applied to pituitary adenomas in different contexts with diverse interpretations. The terms *typical and atypical* adenoma should refer only to pathologic features. *Invasive and non-invasive* to radiological, surgical or morphological findings of invasion, and finally, *aggressive and non-aggressive* to the clinical behavior (69).

## Temozolomide Treatment Outcome

Since 2006, TMZ has been used for the treatment of both aggressive adenomas and pituitary carcinomas. Based on literature search results, to date and to our knowledge, approximately 160 cases have been treated. Numerous publications outlining case reports, retrospective patient studies, clinical practice guidelines, and an international survey have reviewed the outcome of TMZ treatment (39, 66, 70–77). In a recent meta-analysis, the 5-year OS for aggressive pituitary adenomas treated with TMZ was 57.4% and for pituitary carcinomas 56.2% (72). The 5-year progression-free survival was 21.9% for patients with aggressive pituitary adenomas and 36.1% for patients with pituitary carcinomas (72). The mean survival rate of pituitary carcinomas before TMZ was introduced as a treatment option was 1.9 years (78). Hence, TMZ has signified an enormous advancement in the treatment of pituitary tumors (66).

Many parameters may influence response, therefore, with any treatment option, data regarding response rates is of critical importance for clinicians and surgeons. Unfortunately, lack of standardization regarding the criteria to evaluate response rate has generated discrepant results. Some studies consider only complete and partial radiological response as a successful outcome. If the stable radiological disease is included, the response rate for aggressive adenomas varies from 50 to 80.6% and for carcinomas from 50 to 87.6% (72). In aggressive adenomas, TMZ is used as a last resort after various therapeutic options have been exhausted without inhibiting the progression of the tumor. In these cases, as well as in pituitary carcinomas, it should be taken into consideration that a stable disease response may be considered a good response (66).

**Figure 4:**
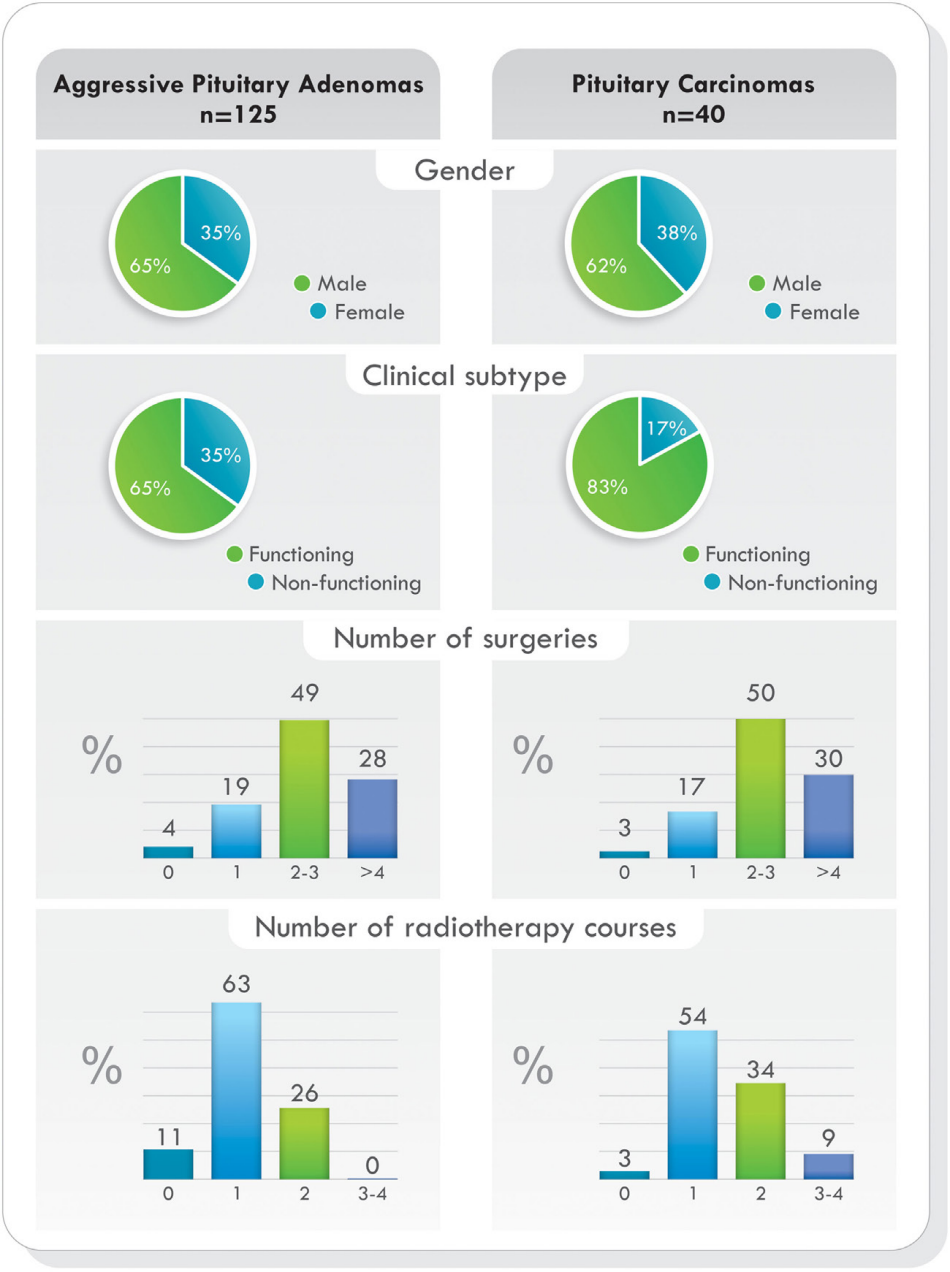
Temozolomide-treated patients. Demographics, clinical presentation, previous surgeries, and radiotherapy comparing aggressive pituitary adenomas and pituitary carcinomas. Data from Ref. (77).

**Figure 5:**
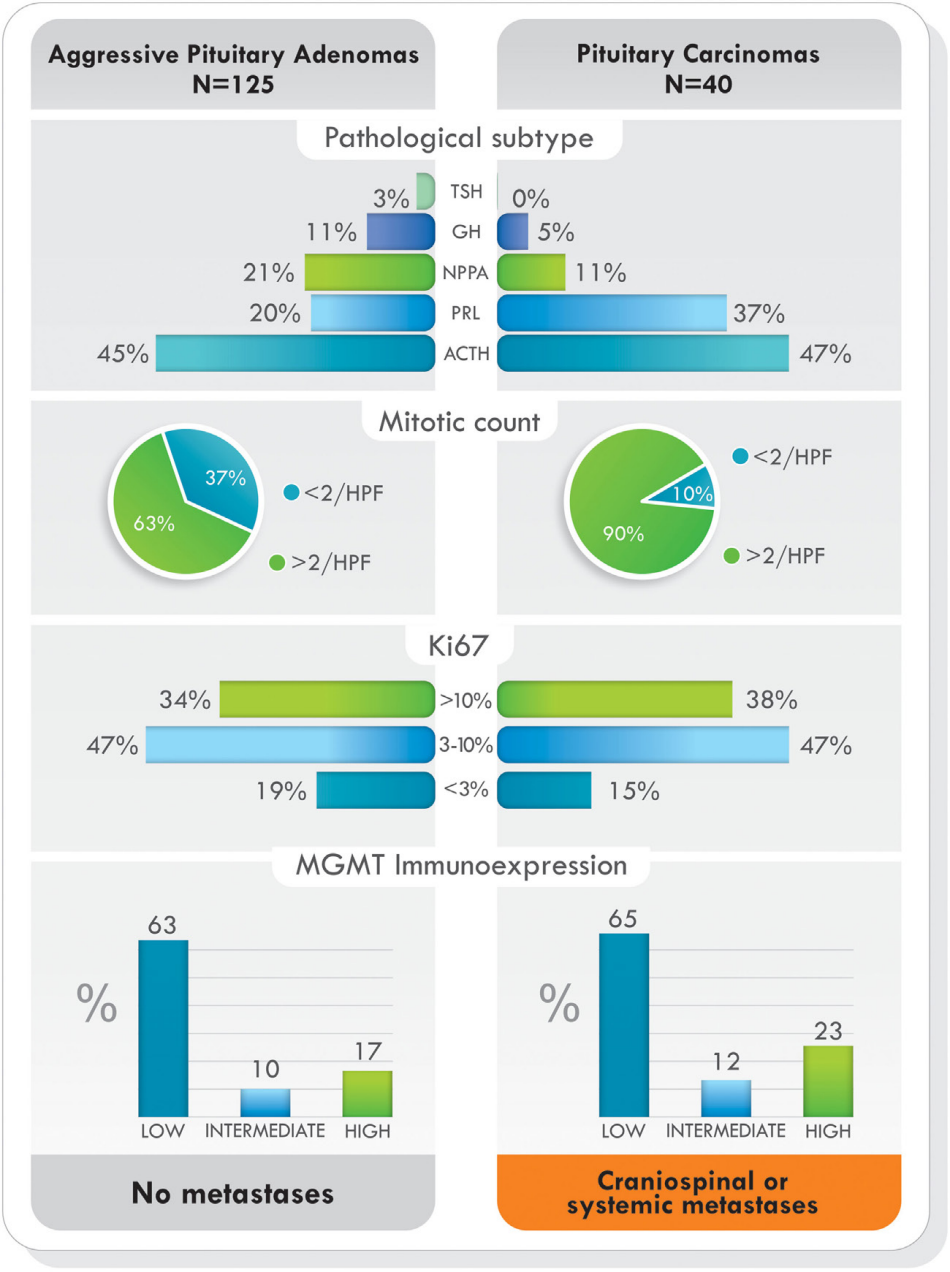
Pathological subtype of tumors, proliferative markers, and O6-methylguanine-DNA methyltransferase (MGMT) immunoexpression in aggressive pituitary adenomas and pituitary carcinomas treated with temozolomide. Data from Ref. (77).

Remarkably, as it was demonstrated in the largest cohort published (77), when comparing demographic characteristics, functional status of the tumors, previous surgeries, previous radiotherapy, and pathological features, no statistically significant differences were found between patients with aggressive pituitary adenomas and pituitary carcinomas. The only difference was the presence of metastases. Data from this study are illustrated in Figures 4 and 5 (77). Due to the lack of morphologic, biochemical, or molecular biomarkers that can indicate in advance which tumors will behave in an aggressive manner, different designations have been used to describe them: premetastatic lesions in the sellar phase (79), carcinomas *in situ* (80), localized pituitary carcinomas (81), and invasive/proliferative tumors with high risk of recurrence—individualizing grade 2b tumors, suspected to be carcinomas without metastases (82). It is possible that some of the tumors diagnosed as aggressive adenomas have already undergone the “malignant switch” and have transformed to carcinomas without recognized metastases. Their identification would be of practical significance because it would bring to light better prognosis and treatment options. To recognize the variable behavior and impact of pituitary tumors on patients, a recent proposal to replace the term pituitary adenoma to pituitary neuroendocrine tumor (PitNET) has been made (83).

The decision to start TMZ in pituitary carcinomas is clear (66, 76) but in aggressive pituitary adenomas other factors such as age, previous medical therapy, radiotherapy, number of previous surgical interventions, invasion, proliferation markers, and histologic subtype of the tumor must be considered (66, 84). The benefits of starting TMZ must outweigh the risks of repeated surgeries, re-irradiation, and potential complications with standard treatments, and in all cases, the decision must be considered from an interdisciplinary perspective (74, 76).

Since studies have shown that pituitary tumors with low MGMT protein expression have had positive response to TMZ (39, 76, 77), it is prudent, but not necessary, to perform MGMT immunohistochemical analysis before starting TMZ therapy (76). If this is not possible, or even if MGMT immunoexpression is high, TMZ can be started for 3–6 cycles to determine the response of the tumor to therapy (76). Standard dosage of TMZ is 150–200 mg/m^2^/day for 5 of every 28 days (5/28). To increase the efficacy of TMZ and tumor response, levels of MGMT should be diminished or depleted. TMZ response is schedule dependent, and alternative dosing regimens may enhance its effectiveness (37, 85). No serious side effects have been reported in TMZ-treated patients with pituitary tumors, and common adverse effects include nausea, vomiting, fatigue, headache, and constipation (66). TMZ is known to affect the formation of sperm in men; thus, couples interested in having children should consider sperm banking before starting treatment. The adverse effects of TMZ and childbearing extend to women as well. Women should not receive TMZ if they expect to become pregnant, are pregnant, or are breastfeeding. In this instance, couples should discuss egg harvesting before treatment initiation.

In TMZ responsive cases, a rapid, early reduction of mass effect has been noted and, in functional tumors, with a remarkable decrease of plasma hormone values (71, 72). As the response can be seen after 3–6 months of therapy, the first imaging evaluation can be performed after three cycles (76). In patients responding to TMZ treatment, cystic change, hemorrhage, tumor necrosis, and shrinkage of the tumor have been seen on MRI (86).

A small number of patients has been treated with a combination of TMZ along with other medications such as capecitabine (87), pasireotide (88, 89), octreotide (90), bevacizumab (91), and thalidomide (76). In patients with functional tumors, the addition of a second medication may be useful. Data regarding these treatment options are limited, and the small number of patients evaluated thus far does not permit an appropriate analysis of these combinations. Their use must be decided according to each case (76).

## Resistance to Temozolomide Therapy

Drug resistance is a significant problem that restricts the effectiveness of chemotherapeutic therapy. Although TMZ remains part of the gold standard treatment for GBM, it is well known that a subset of tumors does not respond to TMZ treatment despite MGMT inactivation. This is an indication that other mechanisms may also modulate the tolerance to TMZ; therefore, overcoming TMZ resistance is a critical matter that must be considered in order to improve treatment outcomes (48).

Currently, it has been shown that MGMT-mediated repair, counteracts the effects of TMZ treatment (19). The key marker of TMZ resistance is the level of gene methylation and protein expression occurring within the cells. Methylation of the *MGMT* promoter occurs in approximately 45% of GBM patients, and it has become a reliable prognostic indicator of TMZ therapy in this type of tumor (92). Contrary to GBM, in pituitary tumors, immunohistochemistry analysis of MGMT has been frequently used, and tumors with low MGMT immunoexpression have generally had a better response to treatment (39, 76, 77, 93). Direct repair by MGMT is the most-documented mechanism associated with TMZ drug resistance, but, apart from the level of MGMT present in cells, other mechanisms may also be involved in acquiring resistance to TMZ. To date, various new molecular mechanisms underlying such resistance are emerging.

In pituitary tumors, low-level immunoexpression of MGMT has been associated with a positive response to TMZ, and high MGMT expression, with lack of response. Nevertheless, some cases with low MGMT expression may not respond to TMZ treatment and a few, with high MGMT could respond (39, 72, 76, 77). Temozolomide produces mainly O6-MeG, N7-MeG, and N3-MeA adducts. The first one is repaired by MGMT (by DR) and the other two by MPG (by BER) (Figure 3). Based on recent findings in GBM, it could be hypothesized that MPG may behave in a similar way in pituitary tumors (42, 43, 94). Hence, pituitary tumors showing *low* MGMT and *low* MPG expression would be highly responsive to TMZ, because unrepaired O6-MeG, N7-Meg, and N3-MeA adducts, would trigger cytotoxicity by different ways (Table 1). Tumors with *high* MGMT expression and *low* MPG could also respond because N7-MeG and N3-MeA adducts, not repaired by BER, would cause cell death. On the other hand, if MPG expression and MGMT were both *high*, the tumor would be highly resistant because all adducts would be repaired. Finally, if the tumor presented *low* MGMT and *high* MPG expression, cytotoxicity or resistance would depend on several factors: an integral MMR pathway, the persistence of mismatch pairing, the tolerance of mutations, and the adequate repair of adducts by BER (Table 1). According to this, in pituitary tumors, it would be advisable to perform immunohistochemical analysis for MGMT as well as for MSH2, MSH6, MLH1, PMS2, and MPG, to evaluate the DNA repair pathways—DR, MMR, and BER, respectively—and the possible response to TMZ therapy (42).

**Table 1:** Response to temozolomide treatment according to immunoexpression of MGMT, MPG, and functional MMR.

	MGMT+	MGMT−	
		
		Adequate MMR	Nonfunctional MMR
MPG+	Resistance	Cytotoxicity?	Resistance?
MPG−	Cytotoxicity	Cytotoxicity	Cytotoxicity

*Based on Ref. (19, 42, 47, 48)*.

*MPG, DNA methylpurine-N-glycosylase; MGMT, O6-methylguanine-DNA methyltransferase; MMR, mismatch repair*.

Tumors may be intrinsically resistant to TMZ before initial treatment; however, resistance may also be acquired during treatment in tumors that were previously sensitive to it. This acquired resistance may be the result of drug-induced genetic changes within tumor cells which impart a survival advantage to certain cells (19). Hence, tumor cells that remain sensitive to TMZ treatment will be eradicated while those that have acquired resistance proceed to divide and produce daughter cells that are also resilient to the effects of TMZ. This acquired resistance may be responsible for TMZ treatment failure in individual patients (19, 95), either because those cells already existed before the onset of treatment as natural phenotypes, or due to the drug-induced mutagenicity. The result is an internal natural selection of genetically heterogeneous clones (96–98).

In GBM cases, it has been shown that TMZ treatment failure may also be the result of an inherent resistance conferred by various mechanisms. It was demonstrated that multi-drug resistance proteins (MRP) intrinsically expressed in gliomas did not respond to chemotherapy (99). Multi-drug resistance proteins along with ABC transporter proteins such as P-glycoprotein 1 (permeability glycoprotein, Pgp) have been shown to cause an increase in drug efflux since they can actively transport various drugs out of the cells (99–101). In this instance, resistance may also be the result of decreased drug influx, although the exact mechanism is still unclear.

The Sonic Hedgehog (SHH) pathway plays a crucial role in neural development. Recently it was found that tumor cells use this pathway to acquire and maintain resistance to TMZ. In studies focusing on GBM, SHH signaling in tumor cells was mediated through an overexpression of the *MDR1* gene which can cause an increase in drug efflux by actively transporting drugs out of cells (99–102).

Another area that may trigger TMZ resistance is the overall mutagenesis—caused by the drug itself—to the entire genomic cellular DNA, impairing, among others, the capacity of tumor cells to repair its DNA. The ability for cells to counter DNA damage is the determining factor of whether these cells are killed by TMZ treatment or if they can become resistant and survive. As mentioned, TMZ treatment causes methylation mainly in guanine but also in adenine. If these adducts remain unrepaired, they will cause replication fork collapse and DNA DSB, resulting in cell cycle arrest (G2/M) and apoptosis (49). For instance, the MMR system plays a significant role in TMZ treatment since it can recognize and remove mispaired bases and insertion/deletion loops produced during DNA synthesis (Figure 3). Therefore, the MMR system must function properly for TMZ to carry out its cytotoxic effects. In a study by Goellner et al., they looked at the relationship between MMR system failure and TMZ resistance in GBM cases (103). Tumors with low-level MGMT expression responded well, initially, to TMZ treatments but concurrently accumulated mutations within the DNA of surviving cells. It was believed that the surviving tumor cells acquired MMR mutations that then resulted in TMZ resistance during further TMZ treatments.

Adaptive response to alkylating agents may have a role in TMZ resistance, especially patients in which TMZ treatment has been discontinued, resulting in tumor progression, and resistance to a second course of treatment (77). The adaptive response to alkylating damage was demonstrated many years ago in studies using *E. coli*. It was noted that *E. coli* exposed to non-toxic doses of alkylating agents, became more resistant to mutations and death. They also became more capable of dealing with higher subsequent doses of the same agent (104–106). This response was also confirmed in human cells (107). Methylating agents triggered the adaptive response in *E. coli* by generating an intracellular signal for its induction (108). If a tumor progresses, tumoral cells may have a similar adaptive response to TMZ, rendering a second course of treatment ineffective (77). In tumor microenvironment MGMT, after its inactivation, may interact with transcription factors of different adaptive response genes, as it has been shown with ROS (109), which induce resistance to subsequent courses of TMZ.

## Future Directions

Molecular drug resistance remains a difficult issue to resolve. It can only be overcome by developing new technologies that allow better characterization of novel signaling pathways, involved in tumor cell response to chemotherapeutic agents. This, in turn, will enable researchers and clinicians to design new treatment regimens that will maximize drug efficacy, while eliminating the mechanism behind drug resistance and minimizing unfavorable health effects.

Temozolomide has shown to be an effective treatment for aggressive pituitary adenomas and pituitary carcinomas, yet some tumors escape its anti-tumor activity. This chemoresistance can be counteracted by targeting several biochemical mechanisms. It has been shown that MGMT is an important TMZ resistance factor. Future studies should focus on developing therapeutic agents that can work in conjunction with TMZ to suppress the effects of MGMT in tumor cells or to attenuate TMZ resistance. For example, an ideal MGMT inhibitor would be a molecule that could specifically dock in the MGMT amino acid sequence (Pro-Cys-His-Arg) that contains the active site (110). Second, novel imidazotetrazine analogs have been recently tested in orthotopic mouse xenografts that showed significantly better brain to plasma ratios compared with TMZ (111). Researchers used imidazotetrazine analogs that acted in the same manner as TMZ yet treatment using these compounds showed lipophilic binding to the entire brain tissue, reducing its availability to the target tumor cells. Nevertheless, chemical modifications are attainable and are under investigation (111). Future research should target the competition of TMZ with the P-glycoprotein (P-gp) which acts as a drug efflux pump that expels the drugs from the cell, reducing its effectiveness in the membrane of tumor cells. Different P-gp inhibitors have been discovered thereby paving the way for an alternative form of combined therapies (112). High levels of MGMT in tumor tissue may be depleted with pseudo-substrates that resemble 06-MeG. O6-benzylguanine and lomeguatrib can deplete MGMT and increase TMZ cytotoxicity (113, 114). Manipulating the BER pathway using methoxyamine can increase TMZ efficacy. To begin the repair process of alkylation, the BER pathway removes the alkylated DNA base, creating an apurinic/apyrimidinic (AP) site which is then repaired. Methoxyamine can form a stable adduct in the AP site, refractory to the downstream members of the BER pathway (46). Future research should focus on developing drug combinations that target various mechanisms. Bevacizumab has been used alone as an alternative in a case of one pituitary carcinoma resistant to TMZ therapy (115). Combined anti-angiogenic therapy along with TMZ treatment could be beneficial, but at this time, the small number of patients treated with this option does not allow proper analysis of the effects of this combination (77). Because of the limited availability of clinical samples and the high cost of performing clinical trials, the use of preclinical *in vitro* human cell line models should be encouraged (116).

The etiology from aggressive to malignant pituitary tumor remains largely unknown. Genomic studies to decipher the driving events are ongoing but are hampered by the limited understanding of the mechanism underlining the tumorigenesis of pituitary adenomas. Novel studies are focusing on changes in microRNAs expression—such as miR-183 and KIAA0101—that result from inhibition of specific transcription factors which would, in turn, lead to overexpression of genes and other molecular events that are upregulated in aggressive tumors. This “aggressive pathway” leads to the activation of cell proliferation responsible for pituitary tumor progression (117). Researchers have also focused on a protein which is involved in estrogen receptor transactivation and metalloproteinase-9 (MMP-9) expression. It was shown that elevated expression of metastasis-associated gene-1 (MTA1) was linked to the aggressive nature of pituitary tumors (118).

A complex network of DNA damage response (DDR) is triggered after damage to tumoral DNA (119). This DDR involves multiple repair mechanisms, cell cycle checkpoints, and tolerance to damage. According to the nature of the DNA injury and the phase of the cell cycle in which the lesion is produced, the cell cycle can be arrested at the G1/S transition, within the S-phase, or at the G2/M transition (119). While potentiation of TMZ treatment can be enhanced by inhibiting the mechanisms behind drug resistance, novel approaches to inhibit the effects of MGMT, BER, and MMR, that invariably arm tumor cells with the ability to combat TMZ activity, must be developed and implemented. Dysfunction of one DNA repair pathway may be compensated for another one, which may contribute to resistance to TMZ treatment in pituitary tumors.

## Conclusion

Temozolomide has shown to be effective in the treatment of aggressive pituitary adenomas and pituitary carcinomas. Overcoming and treating TMZ resistance has been a difficult clinical challenge. Although medical management of TMZ resistance is limited at this time, great strides to understand its underlying mechanisms are being taken. It is evident that arduous challenges persist and that it is crucial that research focus on the development of novel drugs that either enhance the effectiveness of TMZ or overcome its resistance. This will also be beneficial in an attempt for personalized and targeted therapy for pituitary tumors.

## Author Contributions

LS, FR, and KK conceived the study. LS, FR, MC, LO, and CS searched the literature and extracted the data. LS, FR, and MC wrote the manuscript. LO, CS, and KK contributed to the initial revision of the manuscript. All authors contributed to the critical revision of the manuscript before publication and approved the final version.

## Conflict of Interest Statement

The authors declare that the research was conducted in the absence of any commercial or financial relationships that could be construed as a potential conflict of interest.
